# Post-Breeding Season Migrations of a Top Predator, the Harbor Seal (*Phoca vitulina richardii*), from a Marine Protected Area in Alaska

**DOI:** 10.1371/journal.pone.0055386

**Published:** 2013-02-14

**Authors:** Jamie N. Womble, Scott M. Gende

**Affiliations:** 1 Department of Fisheries and Wildlife, Marine Mammal Institute, Oregon State University, Hatfield Marine Science Center, Newport, Oregon, United States of America; 2 Glacier Bay Field Station, National Park Service, Juneau, Alaska, United States of America; Texas A&M University-Corpus Christi, United States of America

## Abstract

Marine protected areas (MPAs) are increasingly being used as a conservation tool for highly mobile marine vertebrates and the focus is typically on protecting breeding areas where individuals are aggregated seasonally. Yet movements during the non-breeding season can overlap with threats that may be equally as important to population dynamics. Thus understanding habitat use and movements of species during the non-breeding periods is critical for conservation. Glacier Bay National Park, Alaska, is one of the largest marine mammal protected areas in the world and has the only enforceable protection measures for reducing disturbance to harbor seals in the United States. Yet harbor seals have declined by up to 11.5%/year from 1992 to 2009. We used satellite-linked transmitters that were attached to 37 female harbor seals to quantify the post-breeding season migrations of seals and the amount of time that seals spent inside vs. outside of the MPA of Glacier Bay. Harbor seals traveled extensively beyond the boundaries of the MPA of Glacier Bay during the post-breeding season, encompassing an area (25,325 km^2^) significantly larger than that used by seals during the breeding season (8,125 km^2^). These movements included the longest migration yet recorded for a harbor seal (3,411 km) and extended use (up to 23 days) of pelagic areas by some seals. Although the collective utilization distribution of harbor seals during the post-breeding season was quite expansive, there was a substantial degree of individual variability in the percentage of days that seals spent in the MPA. Nevertheless, harbor seals demonstrated a high degree of inter-annual site fidelity (93%) to Glacier Bay the following breeding season. Our results highlight the importance of understanding the threats that seals may interact with outside of the boundaries of the MPA of Glacier Bay for understanding population dynamics of seals in Glacier Bay.

## Introduction

Two common objectives for marine protected areas (MPAs) are enhancement of commercial fisheries for sustaining or rebuilding yield, and conservation of biodiversity [1]–[3]. Although the target of biodiversity conservation is often specific habitat features or sensitive ecosystems, increasingly MPAs are being utilized as tools to conserve highly mobile pelagic taxa such as marine mammals, seabirds, and turtles [4]–[8]. For example, the number and diversity of MPAs designated for the conservation of marine mammals is growing globally [9] with increasing calls for more and larger networks of MPAs [10].

Nevertheless, MPA designation can be less than effective in meeting conservation goals for highly mobile marine taxa for a number of reasons. Although the timing and location of the MPAs should correspond to the temporal and spatial distribution of the threat, the actual designation of the MPA boundaries more likely reflects trade-offs between sociocultural, economic, and biological factors [11], [12]. The vaquita (*Phocoena sinus*) provides a good example where a clearly defined population threat such as bycatch [13] can be mitigated with a simple MPA-based solution by expanding existing MPA boundaries [14] but economic constraints prevents its implementation sufficiently to meet the conservation objectives. Additionally, MPAs may not meet species conservation objectives because they may not correspond temporally or spatially with the most pressing threat to the population [15]. For example, many MPAs are established to protect the breeding aggregations of pinnipeds, sea turtles, seabirds, and cetaceans. While important, research demonstrates that major threats may actually occur during post breeding migrations [16] or during dispersal of juveniles [17].

Recognizing that MPAs are a means to a conservation end, monitoring and research for understanding threats and reducing uncertainty into population responses is not just fundamental to understanding MPA effectiveness [18] but also central to conservation efforts for all highly mobile vertebrates [19].

**Figure 1:**
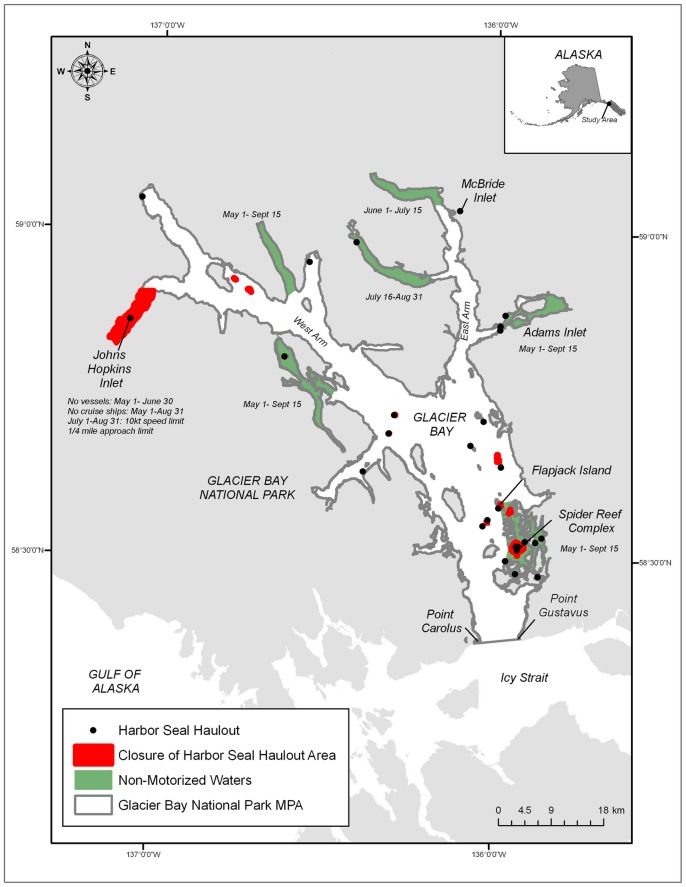
Study site in Glacier Bay National Park, Alaska, United States of America. The map includes harbor seal haulout sites (black circles), closures associated with harbor seal haulout sites (red areas), non-motorized area closures (green areas), dates of closures for each area, and the boundary of marine protected area of Glacier Bay National Park (grey outline). Harbor seals were tagged at the glacial ice haulout site in Johns Hopkins Inlet.

Glacier Bay National Park (Figure 1) is a Biosphere Reserve and World Heritage Site, encompassing over 600,000 acres (242,811 hectares) of marine waters [20]. Although the park was not created solely to protect marine mammals, it functionally serves as the one of the largest marine mammal protected areas in the world [9] with a suite of regulations intended to minimize threats to these species and to sustain a healthy ecosystem for their conservation. For example, regulations require large ships to reduce speed in areas of contemporary and historically high concentrations of endangered humpback whales (*Megaptera novaeangliae*) to reduce the probability of a collision. Glacier Bay is also home to the only enforceable regulations in United States waters aimed at protecting harbor seals (*Phoca vitulina richardii*) from vessel and human-related disturbance [21]. Spatial and temporal regulations for vessels transiting in and near harbor seal breeding areas, and operating regulations once in those areas, are all aimed at reducing impacts of human visitation. Furthermore, subsistence hunting of harbor seals has been prohibited in the park since 1974 [22] and commercial fishing within the MPA boundaries of Glacier Bay is being phased out [23].

By most measures the populations of marine mammals that utilize Glacier Bay are healthy and increasing. Populations of humpback whales using Glacier Bay and surrounding areas are increasing by 5.1% per year [24]. Steller sea lions (*Eumetopias jubatus*) have increased in the Glacier Bay region by 8.2%/year from the 1970’s to 2009, representing the highest rate of growth for this species in Alaska [25]. In addition a Steller sea lion rookery and several haul outs have recently been established in the Glacier Bay region [25], [26]. Sea otters (*Enhydra lutris*), once hunted to extirpation in southeastern Alaska [27] have increased exponentially in Glacier Bay from just a few animals in 1995 to greater than 2,300 in 2004 [28].

In sharp contrast, harbor seals have been rapidly declining [29], [30] despite stable or slightly increasing trends in nearby populations [31]. A suite of recent studies suggest that (1) harbor seals in Glacier Bay are not significantly stressed due to nutritional constraints [32], (2) the clinical health and disease status of seals within Glacier Bay is not different than seals from other stable or increasing populations [33], and (3) disturbance by vessels does not appear to be a primary factor driving the decline [34].

Collectively then, harbor seals in Glacier Bay may be one of the most protected populations of marine mammals in the world, yet the most recent population monitoring data suggest that the declines have not abated or reversed [30]. Here we explore the extent to which harbor seals may be using habitat outside the MPA of Glacier Bay. Although evidence suggests that substantially fewer seals occur in Glacier Bay in late-summer and autumn [35], it is unknown if seals move beyond the boundaries of Glacier Bay, the regions that they may travel to, and the potential threats encountered. Thus, a first fundamental step is to identify movement patterns and habitat use of harbor seals in relation to the boundaries of the MPA of Glacier Bay. Our primary objectives were to quantify the (1) spatial distribution of seals during the post-breeding season (September−April), (2) estimate the utilization distribution of seals relative to the boundaries of the MPA of Glacier Bay, (3) quantify the degree of individual variability in residency patterns of seals in Glacier Bay, and (4) and assess the degree of inter-annual fidelity of seals back to Glacier Bay the following breeding season (May–June).

## Materials and Methods

### Ethics Statement

All harbor seal capture, handling, and research was conducted under Marine Mammal Protection Act (MMPA) permit numbers 358-1787-00 and 358-1787-01 issued to the Alaska Department of Fish & Game and MMPA permit number 782-1676-02 issued to the National Marine Mammal Laboratory by National Oceanic and Atmospheric Administration (NOAA) -Protected Resources Division. Harbor seal capture, handling, and research was also authorized by Glacier Bay National Park under Scientific Research and Collecting permit numbers GLBA-2007-SCI-0003, GLBA-2008-SCI-0004, and associated Glacier Bay National Park and Preserve Waivers to park regulations. Animal use protocols used in this research were reviewed and approved by the Institutional Animal Care and Use Committee at the State of Alaska Department of Fish & Game (protocol 07-16).

### Study Area

Glacier Bay is an estuarine fjord in southeastern Alaska that constitutes a part of Glacier Bay National Park (Figure 1). Distinct oceanographic and circulation patterns [36], [37], as a result of rapid and repeated advances and retreats of tidewater glaciers over the past 225 years [38]–[40], have resulted in sustained levels of mixing, high levels of primary productivity, and abundant communities of forage fish [41], [42]. Johns Hopkins Inlet (58° 50.896' N, −137° 6.121' W), an expansive (12 km long × 2.5 km wide) tidewater glacial fjord in the upper West Arm of Glacier Bay (Figure 1), was chosen as the capture location for seals because the inlet hosts the largest aggregation of seals (>2,000) in Glacier Bay during the summer months and represents one of the primary glacial ice pupping sites for harbor seals in Alaska [29], [30], [43]. In Johns Hopkins Inlet, seals rest upon glacial ice and icebergs that have calved from two advancing tidewater glaciers, the Johns Hopkins glacier and the Gilman glacier.

### Harbor Seal Capture and Instrument Deployment

Juvenile and adult female harbor seals were captured using monofilament nets deployed from inflatable skiffs in September of 2007 (n = 15 seals captured) and 2008 (n = 22 seals captured) (Table 1). Following capture, seals were transported to a research vessel (R/V *Steller*) where they were weighed and curvilinear body length and axial girth were measured. Seal age was determined via morphometrics; seals >3 years old were classified as adults, seals ≤3 years of age were considered as juveniles [44].

**Table 1:** Percentage (%) of grid cells (25 km^2^) that occurred in the 10 (highest intensity), 50, 80, and 100% (lowest intensity) utilization distributions in the marine protected area of Glacier Bay.

Months	Season	Sum of Area (km^2^) of allGrid Cells in 100% UD	10% UD inGB (%)	50% UD inGB (%)	80% UD inGB (%)	100% UDin GB (%)
September–October	Non-breeding	25,325	14	62	40	11
November–December	Non-breeding	22,025	38	44	31	12
January–February	Non-breeding	20,300	38	39	33	12
March–April	Non-breeding	13,975	20	44	34	17
May–June	Breeding	8,125	100	58	41	28

To quantify the spatial and temporal distribution of harbor seals from September through June, we attached satellite-linked transmitters (Spot5, 71.5 mm × 34 mm × 26 mm, 78 g, Wildlife Computers, Redmond, Washington, U.S.A.) to the fur on the heads of juvenile and adult female harbor seals using Devcon 5-minute epoxy adhesive. The instruments were only attached to seals that had obviously completed molting. Instruments were deployed over a 5-day period (11–15 September) in 2007 and over a 7-day period (13–19 September) in 2008. The transmitters were powered by two AA batteries and included a 0.5 w transmitter with a transmission repetition rate of 45 seconds. Conductivity switches inhibited transmissions while seals were in the water and quantified the percent time per hour that the seal was out of water. Instruments were shed during the annual molt which began the following June after capture and varied depending upon the age of the animal, with younger seals molting earlier than older seals [45].

### Track Analysis Using State-Space Models

Locations from each satellite-linked transmitter were estimated by Service Argos (Collecte Localisation Satellite, CLS America, Inc., Largo, Maryland) and downloaded. The Argos locations were filtered with the Douglas Argos-Filter Algorithm v. 7.03 [46] using the following parameters: spatial redundancy (5 km) and maximum sustained rate of movement (10 km/hr).

Following filtering, hourly positions between observed locations were predicted using a continuous-time version of the correlated random walk model (CTCRW) [47]. The CTCRW model incorporates a covariate for Argos location error which is comprised of 6 location classes (Location Class 3, 2, 1, 0, A, B). In addition, a continuously-valued covariate for the percent of each hour spent out of the water for a seal was included in the movement model to account for haulout behavior of seals. The CTCRW model was fit using the Kalman-filter on a state-space version of the continuous time stochastic movement process using the CRAWL package [47] in R (version 13.1). The CTCRW model resulted in an estimate of the most probable track of a seal at hourly intervals, while accounting for Argos location error [47]. Locations that fell on land were removed to establish the final set of locations that were used for subsequent analyses.

### Utilization Distribution of Harbor Seals Relative to MPA of Glacier Bay

Utilization distributions [48] were used to quantify space use of seals relative to the boundary of the MPA of Glacier Bay National Park. A utilization distribution depicts the intensity of use of an area by an animal or a group of animals [49] and is defined as the probability distribution of detecting an animal in given grid cell within a specified time period [50].

The tracks for all seals were pooled to collectively estimate the utilization distribution of seals during the post-breeding season. Utilization distributions for seals were estimated at two-month intervals, or five different time periods, from September through June. The two-month time intervals groupings were based upon similarities in the average distance moved per day per month. Grid cells were chosen as the spatial unit of analysis as they often perform better than other methods, such as minimum convex polygons and kernel density estimation, for quantifying space use of animals [51]. A grid cell size of 25 km^2^ was chosen to allow for detection of individual-scale movements while also providing relatively smooth contours between grid cells [52]. Seal locations were spatially joined with grid cells in ArcGIS and the total number of seal locations per grid cell was summed. The number of seal locations per grid cell was normalized by dividing the number of seal locations per grid cell by the total number of locations for that time period which yielded the proportion of total locations per grid cell for each time period. Proportions of locations per grid cell were sorted from largest to smallest and the cumulative proportions of locations per grid cell were determined to create utilization distributions using custom tools in ArcGIS [52].

The utilization distribution identified the set of all grid cells where a seal location occurred and quantified the probability of detecting a seal in given grid cell within a specified time interval. Grid cells included in the 100% utilization distribution represented areas with the lowest intensity of use by seals whereas grid cells in the 10% utilization distribution represented areas with the highest intensity of use [52]. Utilization distributions of seals for each two-month interval were evaluated with respect to the boundaries of the MPA of Glacier Bay (Figure 1) by estimating the percentage of grid cells that occurred in the 10 (highest), 50, 80, and 100% (lowest) utilization distributions that occurred in the MPA. For the purposes of these analyses, the MPA of Glacier Bay was defined as Glacier Bay proper or all waters inside a line drawn between Point Gustavus (58°2.748′ N, 135°54.927′ W) and Point Carolus (58°22.694′ N, 136°2.535′ W) as all NPS seasonal closures and protection measures focused on harbor seals occur within these boundaries (Figure 1). There were three grid cells that overlapped with the boundary of the MPA of Glacier Bay between Point Gustavus and Point Carolus. If greater than 50% of the area of the grid cell fell inside the boundary of the MPA then the grid cell was considered to be inside the MPA. If greater than 50% of the area of the grid cell fell outside of the boundary of the MPA then the grid cell was considered to be outside of MPA.

### Individual Residency Periods of Harbor Seals in Glacier Bay

The residency periods of individual harbor seals were estimated by determining the proportion of days that a seal spent in Glacier Bay during the post-breeding period, from September through April, using the Douglas Argos-Filter Algorithm (v. 7.03) [46] which selected the best location for each seal per day based on the distance, angle, and rate to the previous and subsequent locations [53]. Residency periods were estimated by plotting the best daily location for each seal in ArcGIS and then assigning each daily location to inside or outside of the MPA of Glacier Bay. The number of days that individual seals spent inside of Glacier Bay was summed to estimate residency periods for individual seals. We only considered seals with complete records, which included seals with tags that transmitted from September through April (n = 27 seals), encompassing 8 months of the post-breeding season.

Multi-response permutation procedures (MRPP), based on a rank-transformed Sørenson distance matrix [54], [55], were used to test for differences in the percentage of days spent by seals in Glacier Bay and the cumulative distance traveled by juvenile and adult seals using PC-ORD [56]. The Sørenson proportional coefficient [57] was used as the distance measure and the *A* test statistic, which ranges from 0 to 1, was reported as a measure of effect size along with the corresponding *p* values.

## Results

Transmitters remained attached to harbor seals for the majority of the post-breeding season (September – April) providing excellent spatial coverage of seal distribution and totaling 8,836 seal tracking days. The average deployment period for satellite-linked transmitters was 238.8 days ±83.7 (SD) (range: 37–335 days) and in some cases transmitters provided location data on individual seals for up 11 months (September to August). Transmitters deployed on adult females ( = 272.4 days ±80.1) (range: 106–335 days) transmitted slightly longer on average than those deployed on juvenile seals (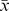 = 224.0 days ±85.4) (range: 37–328 days) likely reflecting the differences in the timing of the annual molt as juveniles molt earlier than adults. 73% (27 of 37) of tags transmitted through May 1^st^ (∼8 months) of the following year after capture.

### Utilization Distribution of Harbor Seals Relative to MPA of Glacier Bay

**Figure 2:**
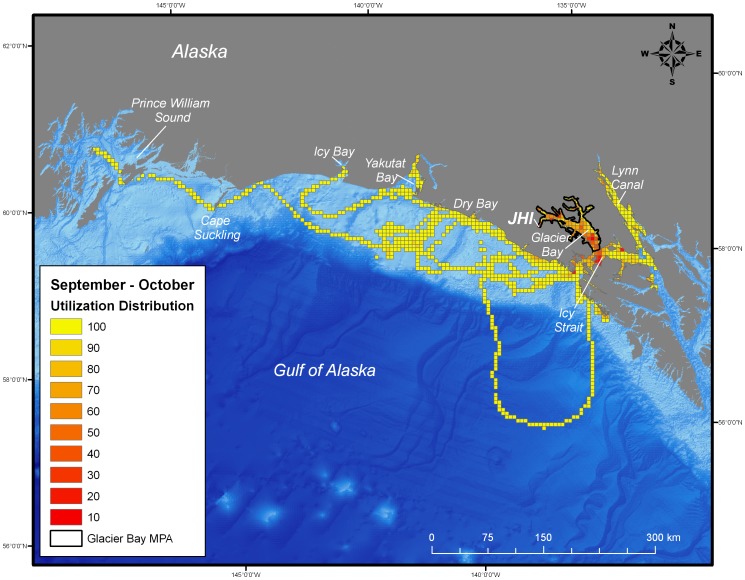
Utilization distribution of female harbor seals (*Phoca vitulina richardii*) during September and October. Boundary of the marine protected area (MPA) of Glacier Bay is shown as black line. JHI indicates tagging location in Johns Hopkins Inlet.

**Figure 3:**
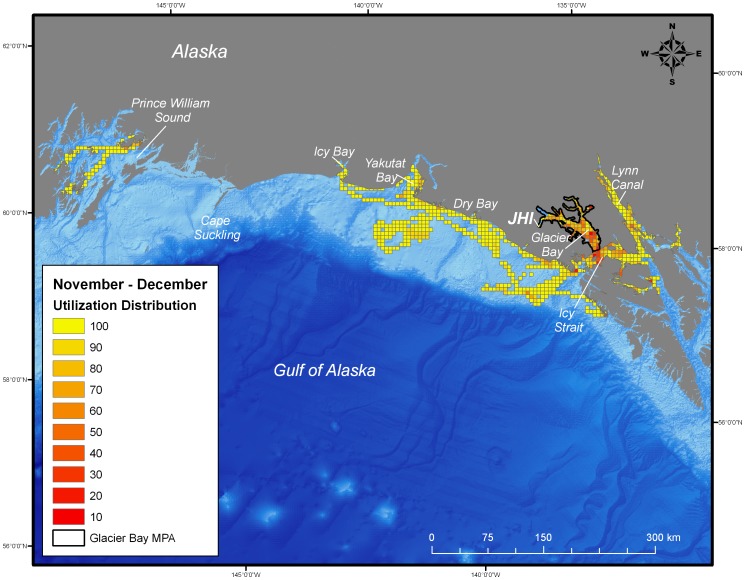
Utilization distribution of female harbor seals (*Phoca vitulina richardii*) during November and December. Boundary of the marine protected area (MPA) of Glacier Bay is shown as black line. JHI indicates tagging location in Johns Hopkins Inlet.

**Figure 4:**
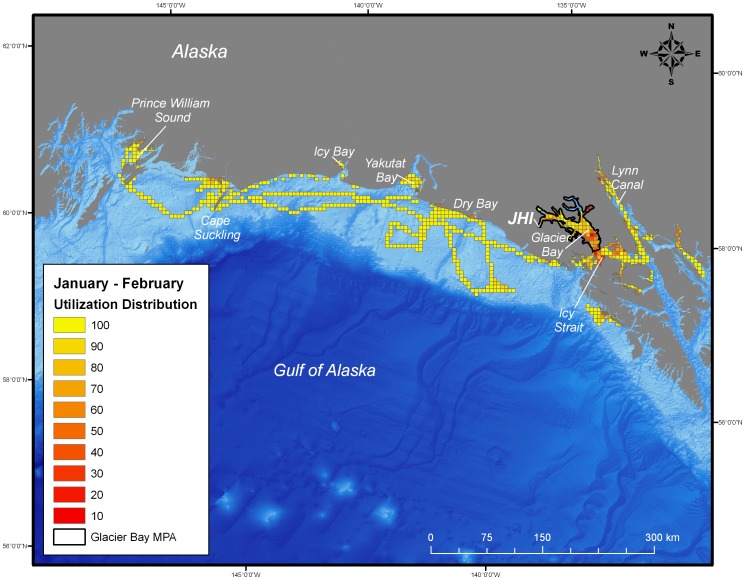
Utilization distribution of female harbor seals (*Phoca vitulina richardii)* during January and February. Boundary of the marine protected area (MPA) of Glacier Bay is shown as black line. JHI indicates tagging location in Johns Hopkins Inlet.

**Figure 5:**
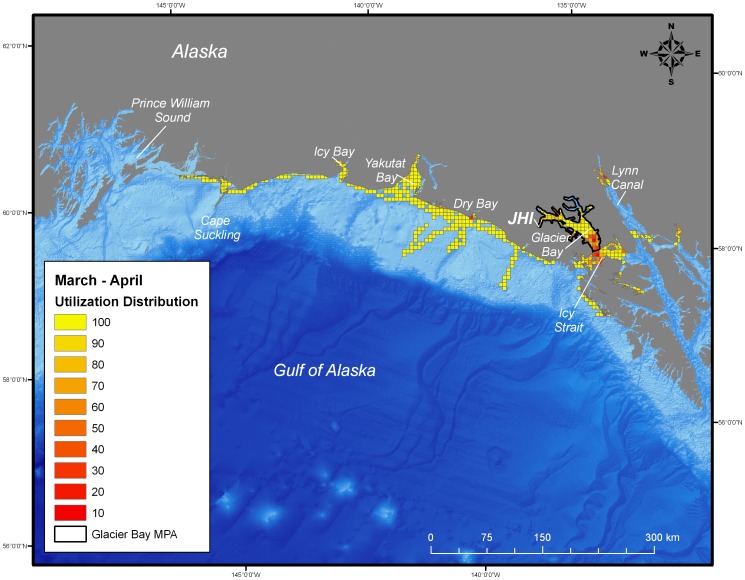
Utilization distribution of female harbor seals (*Phoca vitulina richardii)* during March and April. Boundary of the marine protected area (MPA) of Glacier Bay is shown as black line. JHI indicates tagging location in Johns Hopkins Inlet.

During the post-breeding season, both juvenile and adult female harbor seals ranged extensively both within and beyond the boundaries of the MPA of Glacier Bay. Whereas the glacial ice breeding area of Johns Hopkins Inlet encompasses approximately 22 km^2^, the area used by seals during the post-breeding season encompassed approximately 25,000 km^2^ of which only 2,400 km^2^ was in the MPA of Glacier Bay. Once the seals exited the MPA, they ranged extensively to regions throughout the inside and outside waters of the northern portion of southeastern Alaska and to areas along the continental shelf region of the eastern Gulf of Alaska from Sitka to Prince William Sound (Figures 2, 3, 4, 5). Some seals traveled up to 900 km away (minimum one-way distance) from Glacier Bay to areas in and near Prince William Sound in south-central Alaska. The areas used by seals were primarily restricted to waters along the continental shelf with a few exceptions (Figures 2, 3, 4, 5). Several seals also visited other glacial fjord habitats, including Disenchantment Bay and Icy Bay near Yakutat, during the post-breeding season.

For harbor seals whose tags transmitted from September through April (n = 27), the average cumulative straight-line distance traveled was 2,011 km (±698 SD) (range: 804–3,411 km). The average cumulative distance traveled by juvenile seals (n = 18) was 2,018 km (±501 SD) (range: 1,237–3,239 km) and by adult females seals (n = 9) was 1,198 km (±1,025 SD) (range: 804–3,411 km). There were three seals, one juvenile and two adult females, whose cumulative distance traveled during the post-breeding season exceeded 3,000 km. Differences were not detected in the cumulative distance traveled between juvenile and adult female seals (MRPP: *A* = 0.04, p = 0.07).

**Figure 6:**
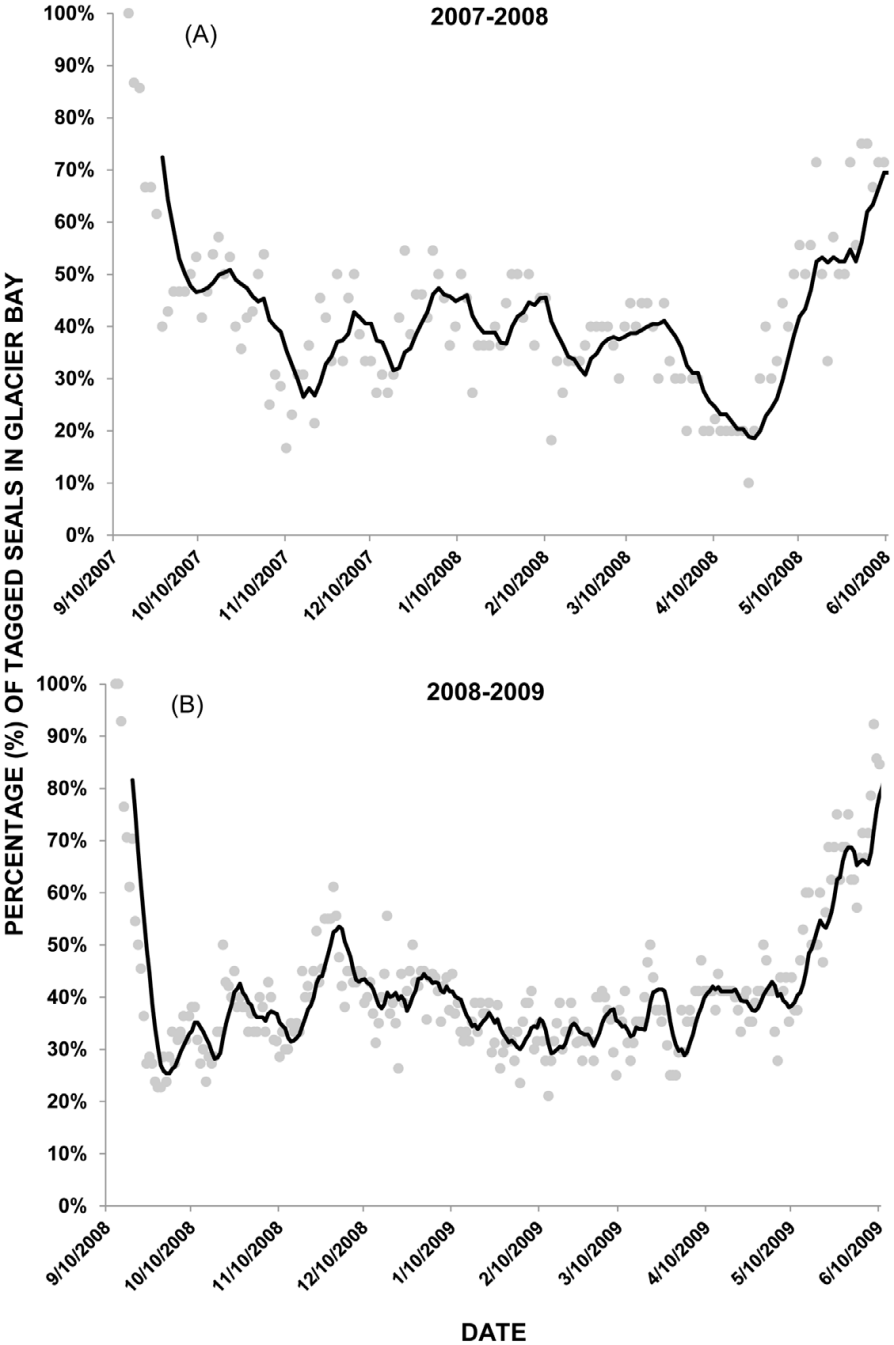
Percentage of tagged harbor seals in Glacier Bay National Park. The percentage of tagged harbor seals in the marine protected area (MPA) of Glacier Bay decreased in mid- to late September in 2007 (A) and in 2008 (B). The percentage of tagged harbor seals in Glacier Bay began to increase starting in late April and early-May in 2008 (A) and in mid-May in 2009 (B).

The percentage of tagged seals inhabiting Glacier Bay decreased substantially in mid- to late September in 2007 (Figure 6a) and 2008 (Figures 6b). The median date of departure of seals from Glacier Bay was 25 September in 2007 (range: 15 Sept to 4 Nov 2007) and 19 September in 2008 (range: 14 Sept to 19 Oct 2008). Tagged seals were largely absent from Johns Hopkins Inlet for extended periods ranging from 27 October 2007 to 22 April 2008 (173 days or ∼5.7 months) and from 6 November 2008 to 2 February 2009 (88 days or ∼3 months). The percentage of tagged seals in Glacier Bay began to increase starting in late April and early-May in 2008 (Figure 6a) and in mid-May in 2009 (Figure 6b).

Collectively, the utilization distribution of seals was most expansive in September and October (25,325 km^2^), November and December (22,025 km^2^), and January and February (20,300 km^2^) (Table 1) demonstrating that some seals ranged extensively from the breeding area in Johns Hopkins Inlet to areas far outside the MPA of Glacier Bay during the post-breeding season (Figures 2, 3, 4). Although the utilization distribution of seals from September through February collectively encompassed an extensive area ranging from northern Southeast Alaska through the eastern Gulf of Alaska and up to Prince William Sound, high-intensity use areas were consistently concentrated in a region spanning from mid-Glacier Bay (inside the MPA) into the adjacent region of Icy Strait (outside of MPA) (Figures 2, 3, 4). A sizeable fraction of the areas most heavily used by seals (the 10% utilization distributions) occurred inside the MPA of Glacier Bay from September through April (Table 1). High-intensity use areas that occurred outside of Glacier Bay were found in Icy Strait, Cross Sound, Lynn Canal, and near Dry Bay along the Yakutat Forelands (Figures 2, 3, 4, 5).

**Figure 7:**
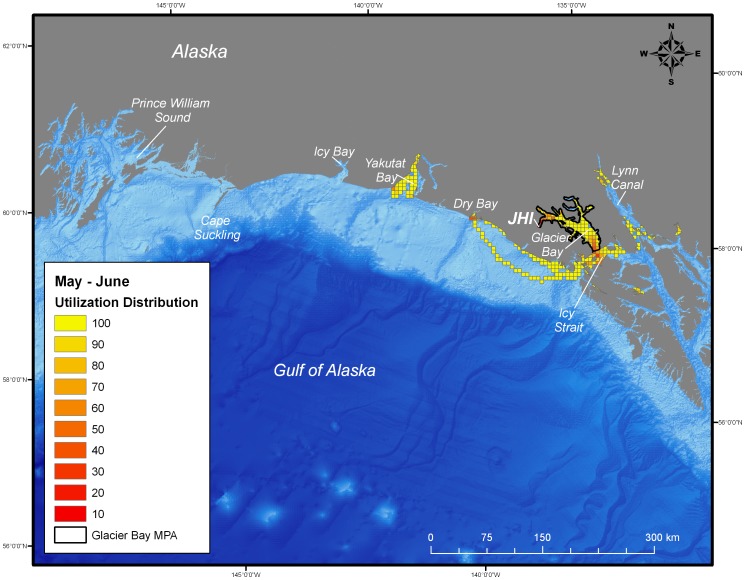
Utilization distribution of female harbor seals (*Phoca vitulina richardii*) during May and June (breeding season). Boundary of the marine protected area (MPA) of Glacier Bay is shown as black line. JHI indicates tagging location in Johns Hopkins Inlet.

In contrast, during the breeding season (May-June) the size of the area used by seals (8,125 km^2^) was substantially reduced and was concentrated primarily in the MPA of Glacier Bay (Figure 7), specifically in Johns Hopkins Inlet. Areas that were used by seals during the breeding season that were outside of Glacier Bay included Disenchantment Bay, a known glacial ice harbor seal pupping site near Yakutat, as well as areas in Icy Strait-Cross Sound, Dry Bay, Lynn Canal, and Taku Inlet (Figure 7).

### Individual Residency Periods of Harbor Seals in Glacier Bay

**Figure 8:**
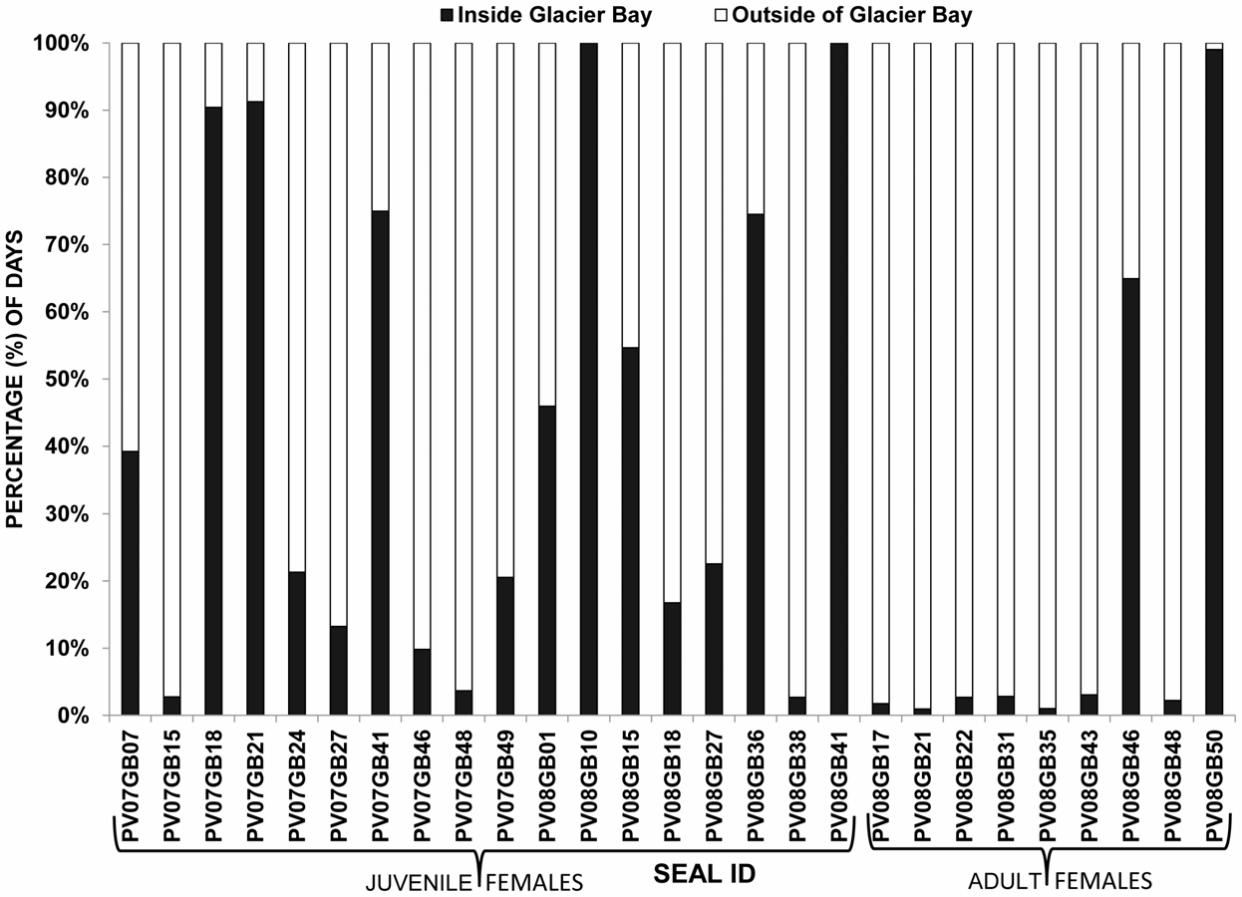
Percentage of days spent inside and outside of the marine protected area (MPA) of Glacier Bay National Park by harbor seals (*Phoca vitulina richardii*) during the post-breeding season. There was a substantial degree of individual variability in the percentage of days that harbor seals spent in the MPA of Glacier Bay. Some harbor seals were more resident to Glacier Bay spending the majority of the post-breeding season inside the MPA whereas other seals were more non-resident spending extended periods of time outside of Glacier Bay.

Although the collective utilization distribution of harbor seals during the post-breeding season was quite expansive, there was a substantial degree of individual variability in the residency patterns or percentage of days that seals spent in the MPA of Glacier Bay. Some seals were more resident to Glacier Bay spending the majority of the post-breeding season inside the MPA whereas other seals were more migratory (Figure 8). Two seals, both juvenile females, spent 100% of time in Glacier Bay and several seals (6 juveniles and 1 adult) spent ≥75% of time in Glacier Bay. Juvenile female seals spent on average 43% (±36% SD) of days in Glacier Bay during the non-breeding season, significantly more than adults (19.8% ±36% SD) (MRPP: *A* = 0.13, p = 0.005) (Figure 8).

There were several seals that exhibited more non-resident behavior and traveled extensively to regions outside of Glacier Bay. Eleven seals spent greater than 90% of days, and 16 seals spent greater than 75% of days outside the MPA of Glacier Bay. Seals that exhibited more non-resident behavior spent extended periods of time in Icy Strait-Cross Sound (4 adults), Lynn Canal (3 juveniles), and the eastern Gulf of Alaska (2 juveniles, 2 adults). In general, once seals arrived at a post-breeding area, they remained primarily in the same region for the majority of the post-breeding period.

**Figure 9:**
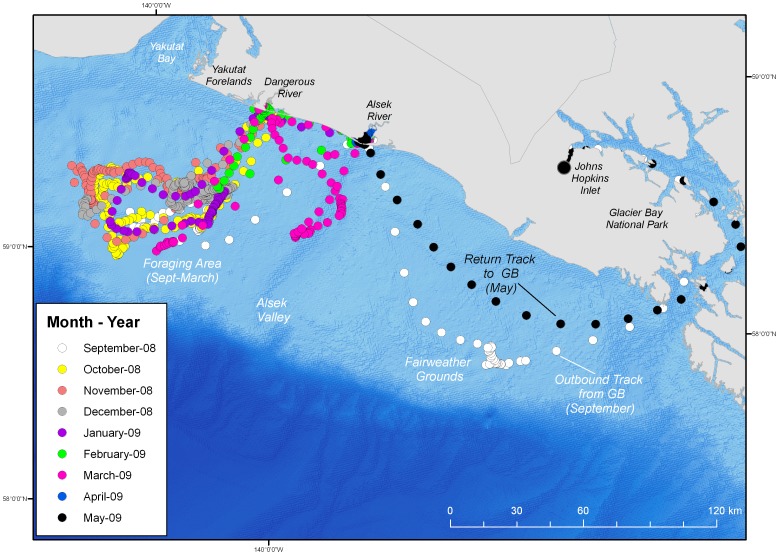
State-space modeled track for adult female harbor seal #PV08GB21. Seal #PV08GB21 spent >200 days in the eastern Gulf of Alaska region and exhibited a high degree of fidelity to a region approximately 95 km from shore on the continental shelf from September 2008 to February 2009. Beginning in late February, PV08GB21 transitioned to a nearshore area at the Alsek River where eulachon (*Thaleichthys pacificus*) aggregate for spawning. Seal #PV08GB21 was tagged in Johns Hopkins Inlet in September of 2008.

Of particular interest were four seals that spent >70% of their time in the eastern Gulf of Alaska along the continental shelf between an area just north of Sitka Sound to Prince William Sound. In the eastern Gulf of Alaska, seals were focused in nearshore areas as well as near the margin of the continental shelf in more pelagic habitat. One adult female seal (PV08GB21) spent >200 days in the eastern Gulf of Alaska region. From September till February, she made several extended forays up to 23 days in length to a pelagic region near the continental shelf margin, approximately 95 km from shore. From late February through mid-May, PV08GB21 transitioned to nearshore areas and exhibited a high degree of fidelity to the Alsek and Dangerous rivers where eulachon (*Thaleichthys pacificus*), an energy-rich forage fish, aggregates for spawning (Figure 9). Similarly, seal# PV08GB22, also an adult female, spent >130 days in the eastern Gulf of Alaska and made repeated visits to the Fairweather Grounds (∼170 kilometers southwest of Yakutat) near the continental shelf margin. PV08GB22 also traveled along the continental shelf to the Copper River Delta and Cape St. Elias just east of Prince William Sound.

### Site Fidelity of Harbor Seals to Glacier Bay

Despite extensive migration and movements of seals away from the MPA of Glacier Bay during the post-breeding season, there was a high degree of inter-annual site fidelity (return rate) of seals to Glacier Bay the following pupping/breeding season (defined as May 1st). For seals with tags that transmitted through May 1^st^ of the year after capture (27 of 37 or 73%), 93% (16 of 18 juveniles; 9 of 9 adults) returned to Glacier Bay and 78% returned to Johns Hopkins Inlet (14 of 18 juveniles; 7 of 9 adults) (Table 2). For those instruments that stopped transmitting before May1st, 80% (8 of 10) were last located in Glacier Bay National Park; however, it is unknown if the instruments stopped transmitting due to instrument failure, instrument loss, or seal mortality.

**Table 2:** Estimates of site fidelity and return rates of harbor seals (n = 37) to Glacier Bay and Johns Hopkins Inlet (JHI) the following breeding season (defined as May 1^st^) after seals were captured.

Year	# of Transmitters Deployed	# of Transmitters working on May 1^st^	# of seals (%) that returned to GLBA	# of seals (%) that returned to JHI
Juvenile Seals	27	18(66.6%)	16(88.8%)	14 (77.7%)
Adult Seals	10	9 (90.0%)	9 (100.0%)	7 (77.7%)
**All Seals**	37	27(73.0%)	25 (92.6%)	21 (77.7%)

## Discussion

Relative to most other MPAs, the size of the MPA of Glacier Bay is extensive (2,400 km^2^) and seals generally stayed within the protected area during the breeding season. In contrast, during the post-breeding season harbor seals traveled extensively beyond the boundaries of the Glacier Bay encompassing an area of approximately 25,325 km^2^. Some harbor seals undertook relatively extensive migratory movements ranging up to 900 km away (one-way distance) to areas in Prince William Sound in south-central Alaska and a few seals traveled cumulative distances exceeding 3,000 km during the post-breeding season. Such extensive post-breeding season migrations and extended use of pelagic areas has not been previously reported for harbor seals from glacial fjords in Alaska or elsewhere and is in contrast to movement patterns of seals reported from most terrestrial breeding areas [58]–[61].

Although seals ranged extensively beyond the MPA during the post-breeding season there was a high degree of inter-annual fidelity (93%) back to Glacier Bay the following breeding season (May-June). Such a high degree of site fidelity is consistent with genetic studies that suggest that philopatry occurs at smaller scales in harbor seals [62], [63]. The high degree of site fidelity of harbor seals to Glacier Bay during the breeding season also supports the recent designation of harbor seals in the Glacier Bay and Icy Strait region as one of twelve stocks of harbor seals in Alaska [64]. Fidelity to breeding sites or philopatry is not uncommon in pinnipeds [65]–[67] or harbor seals [59], [68]–[70] and may confer benefits such as familiarity with local conditions or reduced risk of predation. However, such a high degree of breeding site fidelity may also come at a cost as threats that seals may encounter during the post-breeding season may influence the population dynamics of seals in Glacier Bay.

Most harbor seals traveled extensively; however, there was a substantial degree of individual variability in residency patterns of seals in Glacier Bay. Some seals spent greater than 90% of the post-breeding period outside of the MPA of Glacier Bay whereas other seals remained in Glacier Bay for the entire post-breeding season. Although the relatively high degree of intra-population variation suggests that individual seals may employ different strategies, it is unknown if differences in behavioral strategies of seals may confer fitness advantages. However, populations that exhibit variability in migratory and residency patterns can create challenges for identifying and managing for different behavioral ecotypes [71]–[72]. Ultimately it will be important to ensure that both migratory and resident behaviors are accounted for in the context of designing and monitoring the effectiveness of MPAs.

Although harbor seals are primarily thought to forage in more nearshore shallow coastal areas, some seals exhibited fidelity to more offshore regions. Use of more offshore and pelagic regions near the continental shelf edge suggests that these habitats may be of substantial ecological significance to harbor seals as foraging areas (Figure 9). The presumed foraging trips (n = 5) by seal# PV08GB21 to the pelagic region near the continental shelf-edge were on average 14.4 days in length and ranged up to 23 days. Such persistent use of focal regions by seals and other highly mobile taxa can present opportunities for conservation of important habitats [4]; however, spatial protections are currently limited for pelagic regions and dynamic oceanographic features that are used by highly mobile marine species [73]–[75].

Our study focused only on the post-breeding season migrations of juvenile and adult female harbor seals as females are particularly important in terms of population productivity. However, previous studies have demonstrated that the behavior of male harbor seals may differ from that of females. For example, female harbor seals captured in Prince William Sound, Alaska, typically had larger home ranges than males from September to March [58]. In contrast, during the breeding season, male harbor seals typically traveled greater distances than females in the Pacific Northwest and Scotland [61], [76]. Collectively, these studies demonstrate that sex-specific differences occur and emphasize the importance of understanding and accounting for such differences to ensure effective conservation strategies [75].

The extensive post-breeding season distribution of seals coupled with the high degree of breeding site fidelity to Glacier Bay suggests that a more thorough understanding of the distribution of seals that comprise this stock relative to human-related threats may provide a better understanding of potential factors that may be driving population trajectories in Glacier Bay. However, spatially explicit data regarding human-related threats and the extent to which seals from Glacier Bay interact with these threats are generally lacking.

Commercial and subsistence gillnet fisheries for salmon (*Oncorhynchus* spp.) occur in several areas in southeastern Alaska, including Yakutat Bay, along the coast of the Yakutat Forelands in the eastern Gulf of Alaska, Lynn Canal, and in the Taku Inlet-Stephens Passage area. Many of these areas are also used by harbor seals from Glacier Bay during the post-breeding season; however, the extent to which harbor seals interact with gillnet fisheries in southeastern Alaska is largely unknown. Evidence from other regions of Alaska and from studies elsewhere suggests that gillnet fisheries and their potential impact on pinnipeds may be significant [77], [78]. An estimated 20,867 pinnipeds were caught as bycatch in commercial fisheries in the Pacific Ocean from 1990–1999 and approximately 98% of bycatch of pinnipeds occurred in gillnet fisheries [77]. Similarly, studies in Norway documented substantial interaction between bottom-set gillnets and young-of-year harbor seals [79], [80]. Although there has been limited observer effort associated with marine mammal and gillnet fishery interactions in southeastern Alaska, interactions have been observed in other regions of Alaska [81], [82], suggesting that such interactions may warrant further attention.

Another potential source of mortality is associated with the subsistence harvest of harbor seals by Alaska Natives which is authorized under the Marine Mammal Protection Act. Although subsistence harvest of harbor seals has not been permitted in Glacier Bay National Park since 1974 [22], the extensive post-breeding season distribution of seals from Glacier Bay may expose seals to subsistence harvest outside of the park. Harbor seals are an important cultural and subsistence resource for Alaska Natives, particularly in southeastern Alaska, and harvest has taken place for many generations [83]. Harvested seals are used for meat, oil, skins, and handicrafts as well as for an important item for trading and cultural exchange [83]–[85]. Subsistence surveys and anthropological studies demonstrate that harbor seals may be harvested during all months; however, there are typically two distinct seasonal peaks for harvest of seals which occur during spring and in autumn/early winter [84], [85]. These time periods co-occur with the time period during which seals travel beyond the boundaries of Glacier Bay; however, it is currently unknown whether or not either of these potential threats may have population-level effects on harbor seals in Glacier Bay.

This study advances our understanding of the distribution of a pinniped of conservation concern, the harbor seal, relative to boundaries of one of the largest MPAs in the northern hemisphere. Our results have several implications not only for the conservation of harbor seals in Glacier Bay and other glacial fjord habitats in Alaska but also for evaluating and improving the design of MPAs for other wide-ranging species, such as seabirds, cetaceans, and other pinniped species. First, MPAs are often created in the absence of spatially explicit data for species throughout the annual cycle. The use of discrete areas for breeding and non-breeding activities by highly mobile pelagic taxa highlights the challenges and complexities associated with designing protected areas for species that may inhabit dramatically different regions over the course of the annual cycle [6], [52], [86]. Second, individuals may exhibit a high degree of variability in residency patterns, movements, and migratory behavior thus creating challenges for identifying and managing for different behavioral ecotypes [71]–[72]. Finally, the high-degree of fidelity to breeding areas highlights the importance of understanding the spatial distribution of species of conservation concern throughout the annual cycle as threats encountered during the post-breeding season may influence population dynamics.

Similar to other large marine vertebrates, harbor seals are long-loved and relatively late-reproducing species and these life history characteristics make them particularly sensitive to late-stage or adult mortality [87]. Although the MPA of Glacier Bay provides special protection measures for harbor seals during the breeding season, harbor seals have not recovered. Thus, before firm conclusions regarding the effectiveness of the MPA for harbor seals can be made, it will be important to identify the extent to which harbor seals from the Glacier Bay/Icy Strait stock interact with potential threats and how such threats may or may not impact the population dynamics of harbor seals in Glacier Bay. Similarly, quantifying survival and reproductive rates of seals along with identifying the sources of age-specific mortality [88] for harbor seals both inside and outside of the MPA would also be beneficial.

Our study highlights the challenges associated with managing for highly mobile species that travel in and out of protected areas; however, there are approaches that could be taken to facilitate increased protection for these species. First, a more mechanistic understanding of the relationship between habitat features, prey availability, and the seasonal distribution of highly mobile species is critical and would allow for a predictive approach that could be used to identify features or areas (e.g., seamounts, canyons, eddies, and fish aggregations) where highly mobile species may aggregate. Second, a predictive model of species occurrence relative to habitat features could be coupled with data regarding known and potential threats to predict areas of likely interaction [75], [89]. Coupling these two approaches would provide a mechanistic basis for implementing dynamic time-area closures that could reduce the likelihood of interaction between highly mobile species and potential threats [73], [75], [90]. Finally, increasing the size of MPAs may not necessarily result in complete protection for highly mobile species and also may not be a feasible alternative for a variety of reasons. However, a network of protected areas that collectively encompasses important breeding, feeding, and migratory areas could be a more viable approach that could result in increased protection of highly mobile species throughout much of the annual cycle [6], [75].

Studies of this nature showcase the utility of coupling satellite telemetry and geographic information systems as effective tools for identifying the spatial and temporal distribution of species of conservation concern relative to protected area boundaries [52], [73], [78], [89], [91], which is an important first step in marine spatial planning. Information regarding where species go and the habitats they use is essential for designing protected areas, evaluating the effectiveness of those protected areas, and ultimately for working with stakeholders across jurisdictional boundaries in an attempt to reduce or ameliorate potential anthropogenic threats for species of conservation concern.
